# Characterization and Adaptation of Anaerobic Sludge Microbial Communities Exposed to Tetrabromobisphenol A

**DOI:** 10.1371/journal.pone.0157622

**Published:** 2016-07-27

**Authors:** Emilie Lefevre, Ellen Cooper, Heather M. Stapleton, Claudia K. Gunsch

**Affiliations:** 1 Department of Civil and Environmental Engineering, Duke University, Durham, NC, United States of America; 2 Nicholas School of the Environment, Duke University, Durham, NC, United States of America; Purdue University, UNITED STATES

## Abstract

The increasing occurrence of tetrabromobisphenol A (TBBPA) in the environment is raising questions about its potential ecological and human health impacts. TBBPA is microbially transformed under anaerobic conditions to bisphenol A (BPA). However, little is known about which taxa degrade TBBPA and the adaptation of microbial communities exposed to TBBPA. The objectives of this study were to characterize the effect of TBBPA on microbial community structure during the start-up phase of a bench-scale anaerobic sludge reactor, and identify taxa that may be associated with TBBPA degradation. TBBPA degradation was monitored using LC/MS-MS, and the microbial community was characterized using Ion Torrent sequencing and qPCR. TBBPA was nearly completely transformed to BPA *via* reductive debromination in 55 days. Anaerobic reactor performance was not negatively affected by the presence of TBBPA and the bulk of the microbial community did not experience significant shifts. Several taxa showed a positive response to TBBPA, suggesting they may be associated with TBBPA degradation. Some of these taxa had been previously identified as dehalogenating bacteria including *Dehalococcoides*, *Desulfovibrio*, *Propionibacterium*, and *Methylosinus* species, but most had not previously been identified as having dehalogenating capacities. This study is the first to provide in-depth information on the microbial dynamics of anaerobic microbial communities exposed to TBBPA.

## Introduction

Tetrabromobisphenol A (TBBPA) is currently the most highly produced brominated flame retardant, globally. Primarily used in the electronic industry, TBBPA is added to plastics and printed circuit boards at concentrations ranging from 10 to 20% by polymer weight[1]. Many common household and office products including computers, printers, televisions, coffee machines, and cellphones contain TBBPA[1, 2]. Although not classified as acutely toxic, TBBPA has been shown to illicit various toxicological responses in aquatic wildlife[3–6], and mammals[7]. Likely due to its structural resemblance to the thyroid hormone precursor, thyroxine[8], TBBPA has been shown to disrupt thyroid and estrogen regulative functions[9], cause liver and kidney damage[10], and increase risks of uterine cancer in mammals[11].

Over the last decade, TBBPA has been detected in aquatic sediments, and agricultural soils[12–15] as well as in house and office dust, suggesting that human exposure to this contaminant is likely common[1, 16–18]. In wastewater sludge and waste landfill leachate, TBBPA concentrations as high as 300 ng/g dry weight, and 620 ng/l have been reported, respectively [1, 19–22], suggesting that wastewater treatment effluents and landfill leachates are a likely source for TBBPA to enter natural habitats[13, 17, 23, 24]. The increasing occurrence of TBBPA in environmental samples is raising serious concerns for wildlife and human health. As a result, the last two decades have seen an emergence of studies exploring strategies for TBBPA removal. In particular, strategies based on microbial degradation are cost-effective, and generally accepted to be less intrusive to the environment compared to chemical and physical removal strategies. Although TBBPA mineralization under continuous aerobic conditions can be achieved[25–29], initiating the process by a step in which TBBPA is anaerobically transformed to bisphenol A (BPA) *via* reductive debromination, has proven to substantially increase degradation rates compared to a transformation occurring exclusively under aerobic conditions[30]. Although the anaerobic debromination of TBBPA leads to the generation of BPA, also known as an endocrine disruptor and acutely toxic to aquatic organisms[31], this initial step brings the TBBPA degradation process closer to complete mineralization. Indeed, from there on, rapid mineralization of BPA can be carried out under aerobic conditions, as demonstrated in studies using sequential anoxic-oxic incubations[32, 33]. Additionally, amendments with various carbon and nitrogen substrates have been shown to further increase the rate of TBBPA anaerobic debromination, speeding up the degradation process towards complete mineralization[34].

Microbial communities able to degrade TBBPA are ubiquitous and have been enriched from a wide range of anoxic habitats such as salt-marsh rhizosphere[35], river sediments[26, 33, 36], estuarine sediments[37], soil[32], and wastewater sludge[30, 38]. TBBPA-degrading microorganisms have also been cultured and isolated from these environments[39–43]. However, despite the growing amount of information available, the effect of TBBPA on the taxonomic composition and dynamics of the exposed microbial communities has been overlooked. A few recent studies have used molecular approaches such as 16S rDNA denaturing gradient gel electrophoresis[26, 36, 43] and environmental cloning sequencing[44] to explore community dynamics. However, given the relatively low taxonomic resolution of these techniques, a comprehensive characterization of complex microbial communities exposed to TBBPA is still lacking. In-depth analyses of the whole community are essential, not only for the identification of TBBPA degrading organisms, but also to study how the non-degrading members of the community, responsible for other important ecological processes, respond to the presence of TBBPA. In wastewater treatment systems, as a result of the growing demand for consumer electronics, TBBPA concentrations are expected to increase in the near future. Therefore, understanding how TBBPA affects microbial communities responsible for fundamental wastewater treatment processes, and to what extent TBBPA can be degraded in these systems is extremely relevant to assess the environmental risks associated with the predicted increase of TBBPA concentrations.

The primary aim of this study was to characterize temporal changes in microbial community structure under TBBPA exposure during the start-up phase of an anaerobic sludge reactor. Our second intent was to identify the microbial taxa that may be involved in the TBBPA degradation process. Finally, since both co-metabolic and metabolic pathways have been shown to be involved in halogenated-compound degradation, the effect of sodium acetate, previously shown to substantially increase TBBPA degradation rates[34], was examined as a possible co-metabolic substrate. Liquid chromatography with tandem mass spectrometry (LC-MS/MS) was used to monitor the TBBPA degradation and identify transformation by-products in the reactors. Sludge microbial communities were characterized by performing next generation sequencing of the bacterial 16S rDNA using the Ion Torrent personal genome machine[45] (PGM). Finally, the dynamics of the methanogenic, archaeal, and bacterial populations were examined using quantitative PCR (qPCR).

## Material and Methods

### Chemicals

Tetrabromobisphenol A (TBBPA, 4,4′-Isopropylidenebis (2,6-dibromophenol), 97% purity, CAS 79-94-7) used in the degradation study was purchased from Sigma-Aldrich (St Louis, MO, US). TBBPA stock solutions (0.4 g/l) were prepared in high performance liquid chromatography (HPLC) grade acetone. For LC-MS/MS analysis, TBBPA for calibration standards was purchased from Wellington Laboratories (Guelph, Canada). Bisphenol A (BPA; CAS 80-05-7) used for LC-MS/MS analysis was purchased from AccuStandard (New Haven, CT, US). Mono, di and tri-bromobisphenol A (CAS 6073-11-6, 29426–78–6, and 6386-73-8, respectively) used for LC-MS/MS analysis was provided by Dr. Göran Marsh, (Stockholm University, SE). ^13^C_12_-tetrabromobisphenol A (Wellington Laboratories, Guelph, Canada) served as the internal standard used for TBBPA and lesser brominated BPAs. ^13^C_12_-BPA (Wellington Laboratories, Guelph, Canada) served as the internal standard for BPA. D_8_-BPA (Wellington Laboratories, Guelph, Canada) and ^13^C_12_- 6-hydroxy-2,2',4,4'-tetrabromodiphenyl ether (Cambridge Isotope Laboratories, Tewksbury, MA, US) were used as recovery standards to assess recoveries of ^13^C_12_-BPA and ^13^C_12_-TBBPA, respectively. Solvents used for LC-MS/MS analyses were purchased from Honeywell, Burdick & Jackson Laboratories (St. Muskegon, MI, US).

### Batch reactor operation and sampling

The 12 bench scale anaerobic sludge reactors used in this study consisted of 2 l glass media bottles previously treated for the removal of organic residues in a muffle furnace at 550°C for at least 4 hours. After being autoclaved, bottles were filled with 1.6 l of activated sludge collected the same day from the aeration tank at the North Durham wastewater treatment facility. Bottles were then transferred under an anaerobic workstation where half of the reactors received 5 mM (final concentration) of sodium acetate trihydrate (co-metabolic set), while the other half did not receive any additional source of carbon (metabolic set). Within each set, three replicate reactors (TBBPA-spiked reactors) were spiked with 10 ml of 0.4 g/l TBBPA stock solution for a target final concentration of ~5,000 nM, while the other reactors (control reactors) only received 10 ml of acetone (i.e., solvent used to prepare the TBBPA stock solution). All reactors were hermetically sealed with halobutyl rubber stoppers (Wheaton, Millville, NJ, US), and a plastic cap with a center hole was tightly screwed on top to ensure anaerobic conditions. Reactors were shaken (120 X g) at room temperature (23°C±0.9) in the dark. During the course of the experiment, the volume of gas produced was measured and released from the reactors daily by inserting a needle connected to a glass syringe through the stoppers. Sludge was periodically sampled using an anaerobic workstation using glassware pre-cleaned as described above. Samples of 5 and 3 ml of sludge for chemical and microbial analysis, respectively, were collected from Day 0 to Day 76, every 3 to 15 days depending on the volume of gas produced by the reactors. Over the course of the experiment, a total of 72 ml, which correspond to 4.5% of the reactor initial volume, was sampled. Samples were stored at -20°C and—80°C until processed for chemical and microbial analysis, respectively.

### Chemical analysis

For chemical analysis, 200 μl of sludge was placed in a polypropylene microfuge tube. Each sample set included triplicate blanks (200 μl LC-MS/MS -grade water) and a matrix spike consisting of 200 μl LC-MS/MS -grade water and 100 μl of each of the TBBPA, BPA, and mixed mono, di and tri-bromoBPA calibration stock solutions. Blanks and matrix spikes were processed in microfuge tubes alongside samples. Each sample set included a QA/QC prepared like the matrix spike in a LC-MS vial. All samples, blanks, matrix spike, and QA/QC received 100 μl each of ^13^C_12_-TBBPA and ^13^C_12_-BPA internal standards and 400 μl of methanol. For extractions, microfuge tubes were homogenized for 10 sec using a vortexer, sonicated 5 min in an ultrasonic water bath, and centrifuged for 1 min at 6,000 X g. The supernatant was transferred to a LC-MS vial, and the samples were extracted twice more with 400 μl acetonitrile. Extracts were combined and concentrated to 100 μl under N_2_ and 100 μl of recovery standards (D_8_-BPA, ^13^C_12_-6-hydroxy-2,2,4,4-tetrabromodiphenyl ether) was added to quantify recoveries of primary internal standards. To match starting mobile phase conditions for liquid chromatography, 800 μl LC-MS grade water was added. TBBPA and lesser brominated by-products were analyzed by LC-MS/MS using a Thermo Accela ultra-high pressure LC and Vantage triple quadrupole mass spectrometer. Analytes were separated on a Thermo Hypersil Gold 100 x 2.1 mm column with a methanol-water gradient (80% methanol 0–0.2 min, to 99% methanol at 1.5 min, held at 99% to 3.5 min; 300 μl/min). Analytes were detected by selected ion monitoring (S1 Table). Recoveries of primary internal standards were 80.1–94.0% for ^13^C_12_-TBBPA and 84.0–97.8% for ^13^C_12_-BPA.

### Ion Torrent sample preparation

Microbial community analysis was performed on sludge samples collected at Days 0, 28, and 55. Samples were centrifuged at 8,000 X g for 15 min and total genomic DNA was extracted using the MoBio Power Soil DNA isolation kit (MoBio, Carlsbad, CA, US) from the pelleted microbial biomass. To increase DNA yield, the manufacturer’s protocol was slightly modified. Bead tubes were vortexed for 45 min (step 5 of the manufacturer’s protocol), incubations at 4°C were performed for 20 min (Steps 8 and 11), and 50 μl of molecular water (Sigma-Aldrich, St Louis, MO, US) was used to elute the genomic DNA (step 20). DNA quantification was performed on a Qubit 2.0 fluorometer using the Qubit dsDNA BR Assay Kit (Life Technologies, Carlsbad, CA, US), and for a few samples selected randomly, 2 μl of genomic DNA was separated by electrophoresis on a 1% agarose gel to verify that non-sheared high molecular weight DNA was obtained. The hypervariable V3 region of the 16S rDNA gene (~150 bp) was amplified using the 341F and 518R bacterial primers[46] modified with Ion Torrent adapter and Golay barcode sequences as described in Whiteley et al.[47] (S2 Table). For each sample, 4 replicates of 50 μl-PCR reactions were prepared. The PCR mix consisted of 100 ng of genomic DNA, 200 nM of each primer, 200 μM of each dNTP, 2.5 units of Affymetrix FideliTaq DNA polymerase, 5 μl of 10X PCR reaction buffer provided with the Taq polymerase (Affymetrix, Cleveland, OH, US), and 0.5 mg/ml of BSA. PCR amplifications were performed on a BioRad T100 thermal cycler using the following optimized conditions: an initial denaturation of 2 min at 95°C, followed by 30 cycles of 30 sec denaturation at 95°C, annealing of 1 min at 60°C, and elongation of 1 min at 68°C, and a final elongation of 5 min at 68°C. The PCR reactions were pooled, purified, and concentrated using the NucleoSpin Gel and PCR Clean-up kit (Macherey-Nagel; Bethlehem, PA, US). PCR products were eluted in 35 μl of molecular water and separated by electrophoresis on a 2.3% NuSieve 3:1 agarose (Lonza; Walkersville, MD, US) gel in order to verify if the amplified fragments had the expected size of ~220 bp (including the ~150 bp V3 region and the 69 bp Ion Torrent modified forward and reverse primers) and to eliminate primer excess. Fragments of the correct size were then excised and gel-extracted using the Macherey-Nagel NucleoSpin Gel and PCR Clean-up kit. PCR products were eluted in 50 μl of molecular water and quantified using a Qubit 2.0 fluorometer. For each sample, equimolar amount of PCR product were pooled and gel purified one last time. The sample was then sequenced at the Duke IGSP Genome Sequencing and Analysis Core Facility (Durham, NC, US) on an Ion Torrent PGM sequencing platform with the Ion 316 Chip v2 using the Ion PGM Template OT2 400 Kit (Life Technologies). The version 4.4.1 of the Torrent suite base-calling software was used to generate the read output file.

### Ion Torrent data analyses

QIIME[48] was used to analyze the generated Ion Torrent reads (S1 Fig). Briefly, reads were filtered for quality based on length and mean quality scores. Sequences were clustered together in operational taxonomic units (OTUs) using Uclust[49] with a similarity cutoff of 97%. A total of 5,614,745 raw sequences were obtained using the Ion Torrent PGM. After sequence quality and length filtering, 3,828,606 reads (68%) remained. Because generation of sequencing errors is a well-documented drawback of next-generation sequencing platforms, especially for the Ion Torrent PGM[50–53], an additional filtering step removing OTUs with a minimum abundance threshold of 0.001% was applied (the justification for the selection of this threshold is presented in S2 Fig). This additional filtering step left 3,665,171 reads that clustered into 2,512 OTUs. Community comparison was performed using a weighted UniFrac similarity matrix and analyses of similarity (ANOSIM) were performed in order to test for the effect of time (Day 0 vs. Day 28 vs Day 55), condition (metabolic vs. co-metabolic), and treatment (TBBPA vs. control) on the microbial community composition.

### Quantitative PCR (qPCR)

Three qPCR assays targeting the *mcrA* gene[54] (*mcrA*_1035F-*mcrA*_1530R primer set), archaeal 16S rDNA[55] (ARC_787F/ARC_1059R primer set), and bacterial 16S rDNA[55] (BAC_338F/BAC_805R primer set) were performed. The 20 μl-qPCR reactions consisted of 20 ng of genomic DNA, for *mcrA* and archaeal 16S rDNA assays, and 0.04 ng for the bacterial 16S rDNA assay, 200 nM of each primer and 1 X of the iTaq Universal SYBR Green Supermix (BioRad Laboratories, Hercules, CA, US). PCR amplifications were performed on the Stratagene Mx3000P QPCR System (Agilent Technologies, Santa Clara, CA, US) using the following optimized conditions: An initial denaturation of 2 min at 95°C, followed by 40 cycles of 30 sec denaturation at 95°C, annealing of 30 sec at 60°C, and elongation of 30 sec at 68°C. A melting curve analysis, consisting of a gradual increase of temperature from 55 to 95°C, was immediately performed following each assay, and confirmed the absence of primer dimers and non-specific PCR products. Purified pCR2.1-TOPO vectors containing *mcrA*, archaeal 16S rDNA, or bacterial 16S rDNA target regions were used to generate standard curves for each assay. These plasmids were obtained from a previous study conducted in our laboratory on anaerobic reactors (unpublished), in which *mcrA*, archaeal 16S rDNA, and bacterial 16S rDNA PCR products were amplified using the conditions used in this study, and cloned using the TOPO TA cloning kit with TOP10 chemically competent cells following manufacturer’s instruction (Life Technologies). Results were expressed in number of copies per ml of sludge and a series of t-tests were performed to detect significant (p≤0.05) differences between the overall mean of samples grouped by time point, and between means of samples within each time point.

## Results and Discussion

### TBBPA degradation

**Fig 1:**
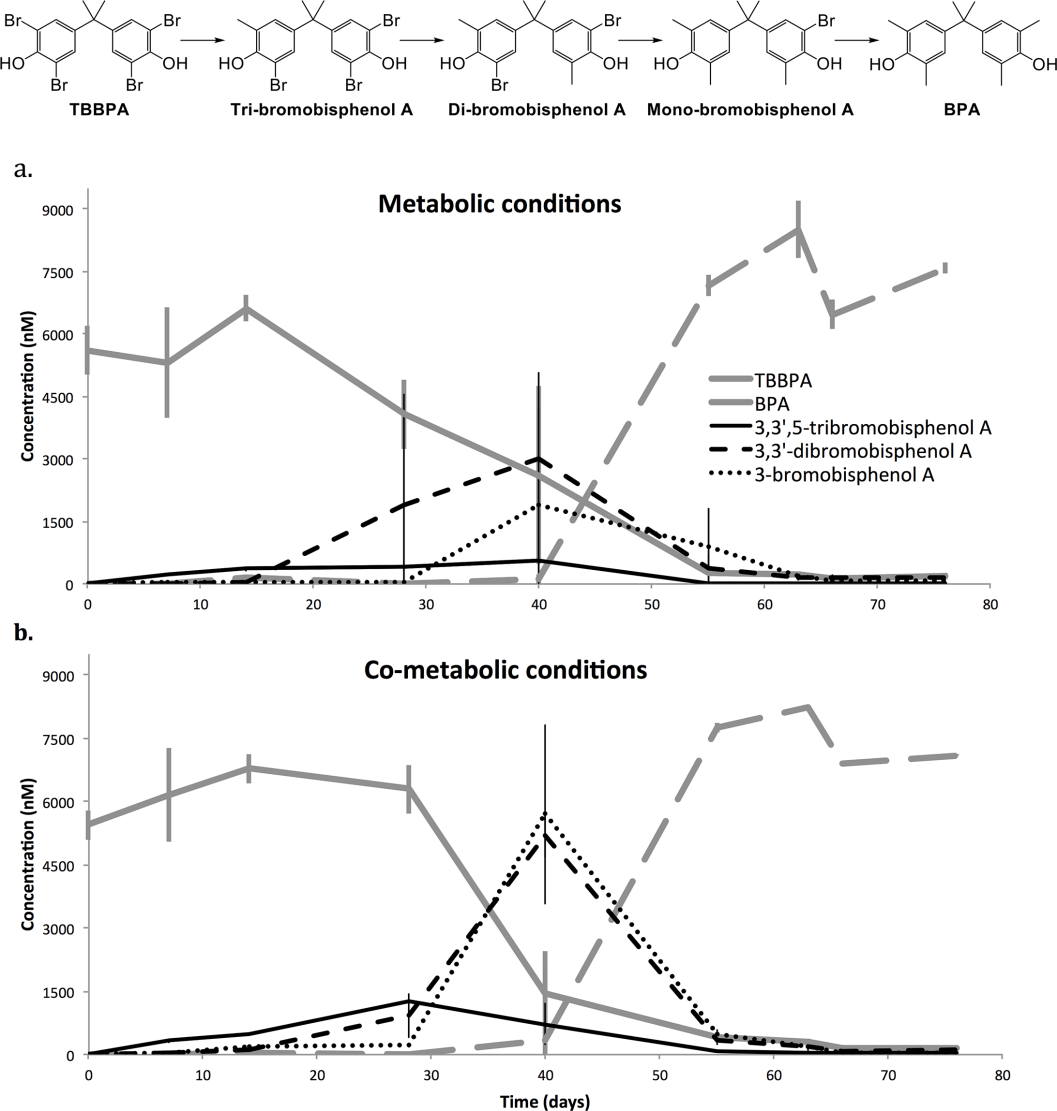
TBBPA degradation and formation of BPA. Concentration of TBBPA, BPA, and degradation by-products (i.e., 3,3',5-tribromobisphenol, 3,3'-dibromobisphenol and 3-bromobisphenol A) in metabolic (a) and co-metabolic (b) reactors overtime. Error bars represent standard deviation from the mean. TBBPA reductive debromination pathway is shown above the graphs.

Concentrations of TBBPA and by-products in the sludge reactors were monitored over 76 days (Fig 1). An initial TBBPA concentration of 5,530±436 nM was measured in the spiked reactors at day 0. In the control reactors, the average background TBBPA concentration was 1.29±7.17nM over the course of the experiment. At Day 28, while no significant degradation had occurred in the co-metabolic reactors, 26±21.6% of TBBPA had already been transformed in the metabolic reactors. At day 40, however, 72.6±14.3% of the spiked TBBPA had been transformed in the co-metabolic reactors, compared to only 47.1±36.8% in the metabolic reactors. Therefore, although TBBPA degradation started after a longer lag period in the co-metabolic reactors, TBBPA was almost completely transformed over a shorter period of time (between day 28 and day 40). In comparison, in the metabolic reactors, the same level of TBBPA degradation was achieved over a much longer period of time (between day 14 and day 55), suggesting that the presence of sodium acetate initially delayed TBBPA degradation but later on, increased TBBPA degradation rate. Replicates of TBBPA-spiked metabolic reactors showed noticeable differences in TBBPA degradation performance, as shown by the standard deviations reported, especially at day 28 and 40 (Fig 1). These observed differences in TBBPA degrading performance between replicate reactors are likely the result of stochastic factors (i.e., stochastic end of dormancy stage, colonization, and extinction of microbial species) that can occur in a small volume system such as the bench-scale reactors used in this study, and have an impact on species assembly processes, particularly during the start-up phase[56]. 3,3',5-tribromobisphenol (tri-BBPA), 3,3'-dibromobisphenol (di-BBPA), and 3-bromobisphenol A (mono-BBPA) were the only degradation by-products detected in the TBBPA-spiked reactors during the course of the experiment, suggesting that the main degradation pathway occurring in all reactors was a reductive debromination of TBBPA to BPA (Fig 1). Differences in the dynamics of these by-products could be noted between reactor conditions, however. At day 28, while no significant degradation had yet occurred in the co-metabolic reactors, 26±21.6% of TBBPA had already been transformed in the metabolic reactors, and di-BBPA was the dominant by-product (representing ~80% of all degradation by-products). At day 40, however, 72.6±14.3% of the spiked TBBPA had been transformed, and di- and mono-BBPA were equally represented in the co-metabolic reactors. Therefore, although the reported results cannot fully explain the observed differences in TBBPA degradation between co-metabolic and metabolic reactors, it is likely that sodium acetate had an effect on the rate of some of the TBBPA debromination steps of the pathway described here. By day 40, no BPA had been detected in any of the reactors. After 55 days of operation, however, when 93.7±1.6% of the spiked TBBPA had been degraded, BPA was detected in all TBBPA-spiked reactors at an average concentration of 7455.6±486.5 nM. This concentration was surprisingly higher than the initial TBBPA concentration spiked. It is unlikely, however, that this difference is a result of compound-specific extraction efficiency variation as a specific mass labeled internal standard was added prior to extraction for each compound. Rather, we attribute this difference to the degradation of BPA-containing parent compounds other than TBBPA which have been shown to also be present in the sludge[57]. Background BPA concentration in the control reactors was 5.6±31.3 nM during the course of the experiment. No further BPA degradation was observed, which is common under anaerobic condition[26]. Others have suggested that the presence of a methylene linker joining the two aromatic rings of the BPA molecule prevents its degradation by anaerobic microorganisms[26]. Although sodium acetate had previously been showed to increase TBBPA degradation[34], it delayed TBBPA transformation in our study, suggesting that if co-metabolic degradation occurred, the sludge organic load was supplying enough electron donors for the co-degradation to proceed, or that sodium acetate was not the most suitable substrate for the present community.

### Bacterial community α-diversity

After the read filtering workflow was applied, a total of 2,512 OTUs were obtained, which is in the range of what previous studies using next-generation sequencing platforms on sludge samples have reported[58–60]. Chao1 individual-based rarefaction curves (S3 Fig) indicated that the microbial communities were well sampled, allowing for diversity comparisons without risks of misinterpreting the results and drawing incorrect conclusions. This was confirmed by the Good’s coverage values (S3 Table), which were above 99% for all samples. Shannon indexes ranged from 6.39 to 7.88, which again is similar to what others have measured in sludge samples[59, 60]. In order to compare the α-diversity between samples grouped by factors (i.e., Day: 0, 28, 55; Treatment: TBBPA, control; condition: metabolic, co-metabolic) a series of t-tests was performed (S4 Fig). While no differences were detected between treatments and conditions, samples collected at Day 28 were significantly (p≤0.05) more diverse than the rest of the samples. Evenness was close to 0 for all samples (S3 Table), indicating that only a few OTUs dominated the community. Indeed, 5 out of 2,512 OTUs accounted for up to 30% of the total community. Overall, OTUs affiliated within 39 phyla, 99 classes, 181 orders, 307 families, and 500 genera. The dominant phyla were Actinobacteria (37.63%), Proteobacteria (32.25%), and Bacteroidetes (12.83%), followed in lesser proportions by Chloroflexi (3.43%), Firmicutes (3.24%), the candidate division TM7 (2.64%), Acidobacteria (2.41%), and Chlorobi (2.20%; S5 Fig). Although these phyla are commonly represented in wastewater sludge[44, 58, 59, 61], the proportion of Actinobacteria is usually below 1% [44, 59, 61]. In this study, the unusually high proportion of Actinobacteria detected might be indicative of a recent sludge-bulking event. Indeed, well-known bulking and foaming bacteria, including *Mycobacterium* sp., *Gordonia* sp., and *Microthrix parvicella*, represented nearly 6% of the total community in our samples[62–64].

### β-diversity

**Fig 2:**
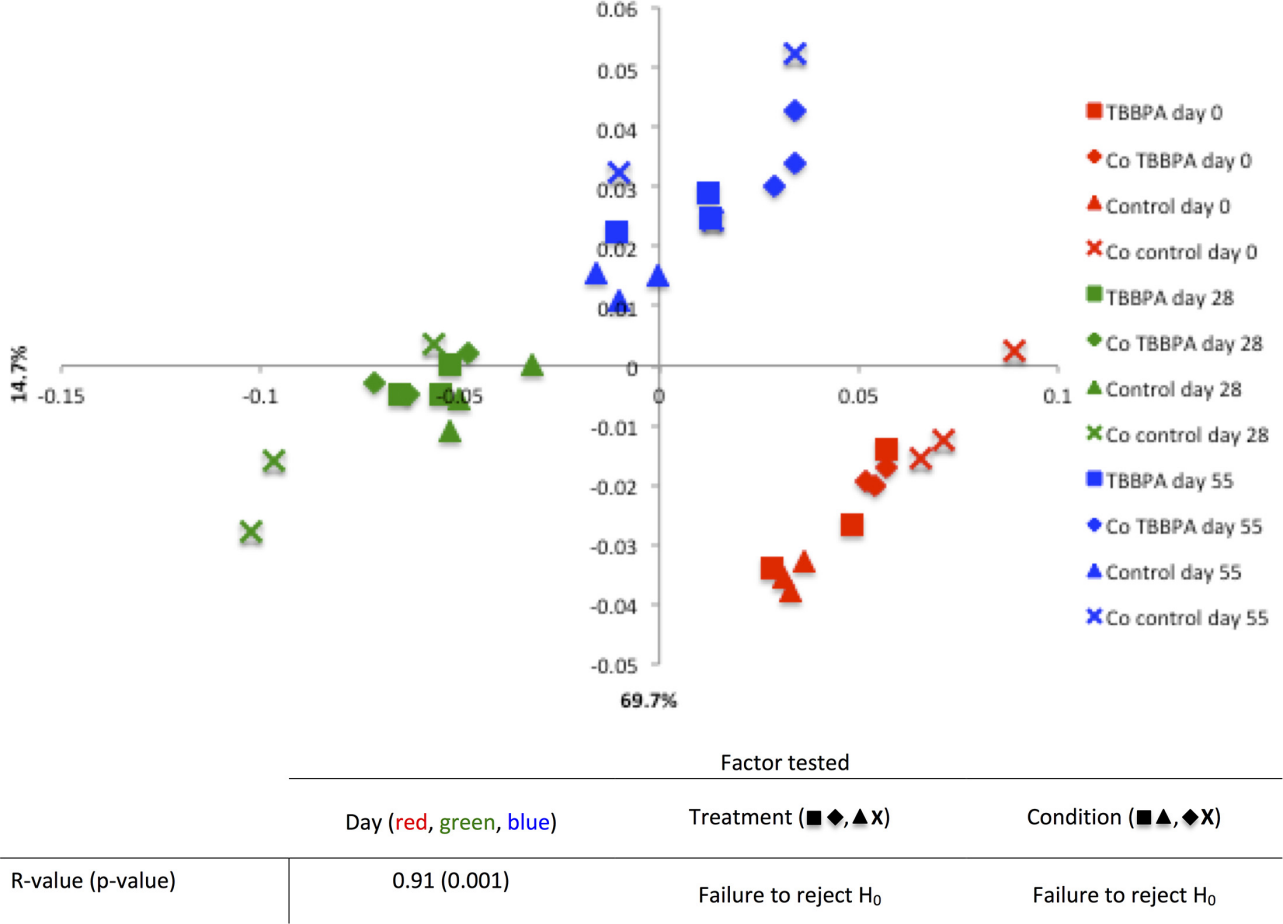
Weighted UniFrac matrix-based Principal Coordinate Analysis (PCoA) and Analysis of Similarity (ANOSIM) results. The percentage of variation explained for the x and y-axis are indicated on the graph. The table indicates the results of the ANOSIM analyses performed on the weighted UniFrac matrix generated. The null hypothesis (H_0_) states that there is no difference between groups in terms of community composition. H_0_ is rejected if p>0.05. An R-value close to 1 indicates an important differences between the groups tested, while an R-value close to 0 indicates a small difference between the groups tested in terms of community composition.

The genus-level taxa relative abundance of the microbial community for each sample is shown in S6 Fig. In terms of taxonomic richness, the microbial communities did not seem to experience drastic changes over time. All reactors regardless of sampling day, treatment or metabolic conditions shared over 96% of their OTUs. In terms of OTU relative abundance, however, notable differences could be observed between sampling days. The community composition shifted from Day 0 to 28, but returned closer to its initial structure by Day 55. This shift appears to have been driven by the most abundant taxa, which, at Day 28, had their lowest abundance, while rare taxa had a slightly higher relative abundance (S6 Fig), which explains the significantly higher Shannon diversity calculated for Day 28-samples (S4A Fig). In order to elucidate additional patterns between treatments and conditions, weighted UniFrac matrix-based PCoA analyses and analyses of similarity (ANOSIM; Fig 2) were performed. The PCoA clearly clustered samples by day, suggesting that communities sampled the same day were more similar to each other than they were to any other communities sampled any another day. The R-values generated by the ANOSIM fell close to 1 when the factor ‘day’ was tested, again indicating that time was the main force driving microbial community dynamics. Although the addition of sodium acetate delayed the degradation of TBBPA (Fig 1), it did not have an effect on the overall microbial community composition. Sodium acetate might have negatively impacted TBBPA degraders or bacteria tightly interacting syntrophically with them at the beginning of the experiment, delaying TBBPA biodegradation. However, these TBBPA-degrading microbial consortia might represent only a small proportion of the community present in the bioreactors, explaining why no changes in the overall microbial community could be detected[65]. When treatment was tested, no significant differences between groups were found either, suggesting that the presence of high concentrations of TBBPA, and BPA at the beginning and end of the experiment, respectively, did not substantially influence the microbial community dynamics overall. Given that the concentration of TBBPA used in this study is an order of magnitude higher than some of the highest concentration measured in environmental sludge samples, this is quite an unexpected outcome. This is particularly surprising given the very low evenness of our community and that greater evenness has been shown to be associated with greater stability of microbial communities[66]. Even more surprising is that this study, rather than focusing on a steady state anaerobic reactor, where microbial community are well-established and presumably more stable, looked at the start-up phase, during which microbial communities are still shaping to new and changing environmental physiological conditions[67]. These findings, although unexpected, are somewhat reassuring in that microbial communities are able to withstand high concentrations of TBBPA and BPA, suggesting that sludge microbial communities are highly resistant to such a disturbance, but also functionally flexible in that the overall community composition was maintained while TBBPA was degraded[68].

### Methanogenic, archaeal, and bacterial population dynamics

**Fig 3:**
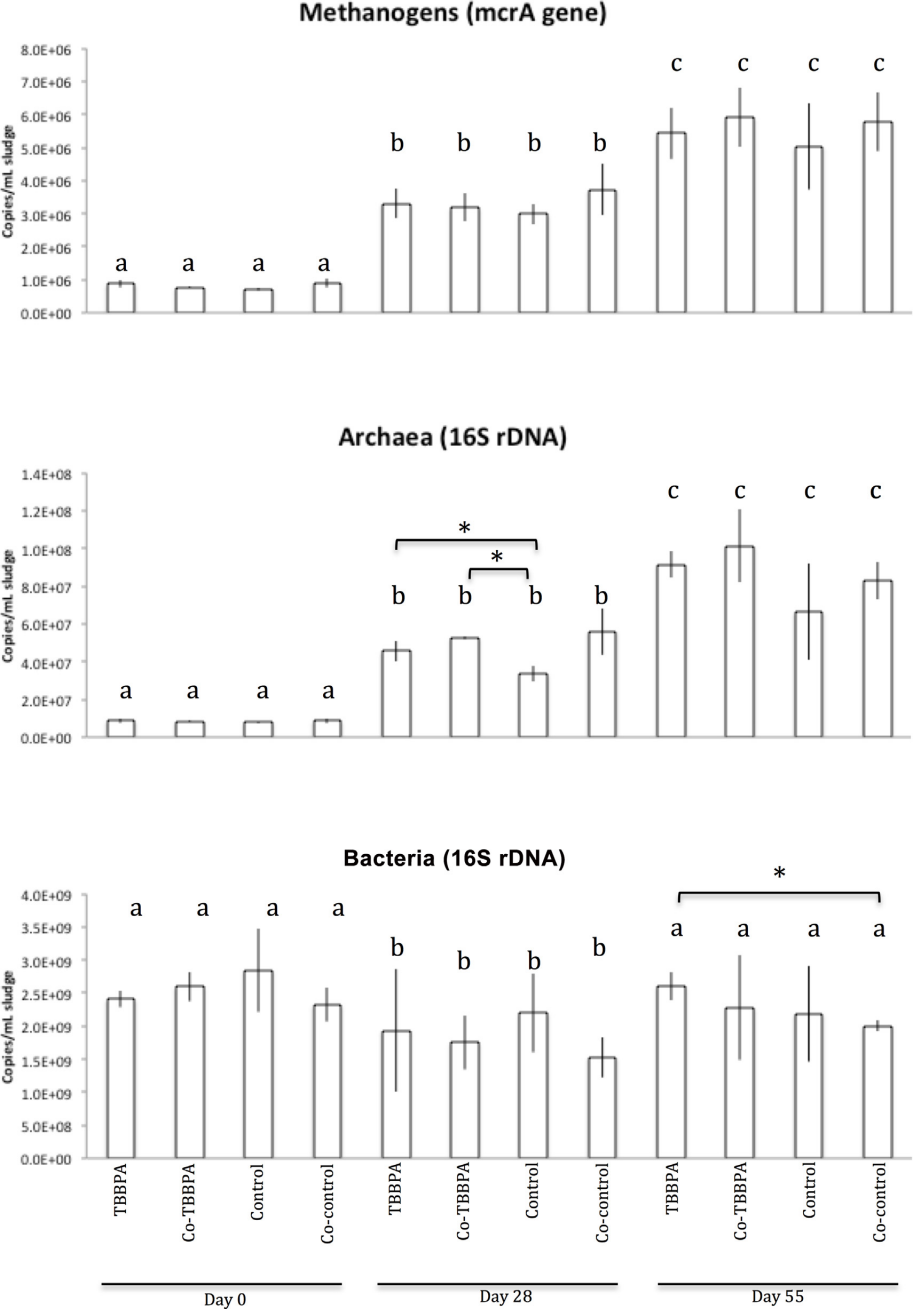
Dynamics of methanogenic, archaeal, and bacterial populations. Quantification of the *mcrA* gene, archaeal 16S rDNA, and bacterial 16S rDNA as measured by qPCR. Efficiency and R^2^ calculated from the standard curves were 89.7% and 0.911, 86.7% and 0.982, and 88.3% and 0.998, for the *mcrA*, archaeal, and bacterial 16S rDNA assays, respectively. Error bars represent the standard deviation from the mean. Different letters above bars indicate significant differences, according to a t-test (p ≤0.05), between days. If a bracket and an asterisk are present between two bars, it indicates that the two corresponding samples are significantly different, according to a t-test (p ≤0.05), performed within each day separately.

qPCR was used to monitor the temporal dynamics of methanogens, Archaebacteria, and Eubacteria in our reactors (Fig 3). The methanogenic population was targeted using the *mcrA* gene, which encodes for the alpha subunit of the methyl coenzyme reductase, which catalyzes the final step of methanogenesis. Because *mcrA* gene abundance strongly correlates with methane production in anaerobic digesters[69, 70], it has been proposed as a key indicator of the performance of anaerobic biodigesters[71]. At Day 0, *mcrA* copy numbers were relatively low and likely corresponded to the non-active archaeal community of the sludge used to seed our reactors[72]. Indeed, although the setup of the reactors occurred under anaerobic conditions, it is most likely that oxygen was still present in the sludge at the beginning of the experiment, initially inhibited the metabolic activity of methanogens. At later days though, *mcrA* copy numbers significantly increased, indicative of an increase in methanogenic activity, which was likely associated with the progressive disappearance of oxygen in the reactors, as expected during the start-up phase of anaerobic reactors. No significant differences between TBBPA-spiked and control reactors were detected using this assay. The archaeal 16S rDNA assay revealed the same temporal pattern as the one observed using the *mcrA* assay. Unlike the *mcrA* assay however, the archaeal 16S rDNA assay revealed significant differences in copy number between the TBBPA-spiked reactors and metabolic controls at Day 28. Because the archaeal community in anaerobic digesters is likely to be dominated by methanogens[55], both assays were expected to give the same population dynamic pattern. However, Wilkins et al.[73] who sequenced these two genes from the same anaerobic digester obtained substantially different taxonomic profiles, suggesting that the primer sets used for each assay are biased towards certain methanogen taxa. Therefore, our 16S rDNA assay may have amplified methanogenic taxa that the *mcrA* assay did not detect, and that may indirectly benefit from the addition of TBBPA or sodium acetate. Iasur-Kruh et al.[36] who examined the role of methanogens in TBBPA degradation found that when methanogenesis was inhibited, TBBPA degradation still occurred but was slightly delayed, suggesting a minor but beneficial role of methanogens in the TBBPA degradation process. In the present study, methanogens might have indirectly benefited from the metabolism of TBBPA-degraders. The ratio between the overall archaeal and bacterial 16S rDNA copy number indicated that the archaeal population represented ~2% of the total prokaryotic population, which is in the range of previously reported values in anaerobic digesters[58]. The bacterial 16S rDNA qPCR assay showed a different temporal pattern. The abundance of the overall bacterial community significantly decreased from Day 0 to Day 28, then, at Day 55, returned close to its initial abundance. However, except for Day 55, no significant differences between treatments were observed. This may be due to the low sensitivity of our assay as indicated by the high variation of the measurements (Fig 3).

### Identification of TBBPA-degrading taxa

**Fig 4:**
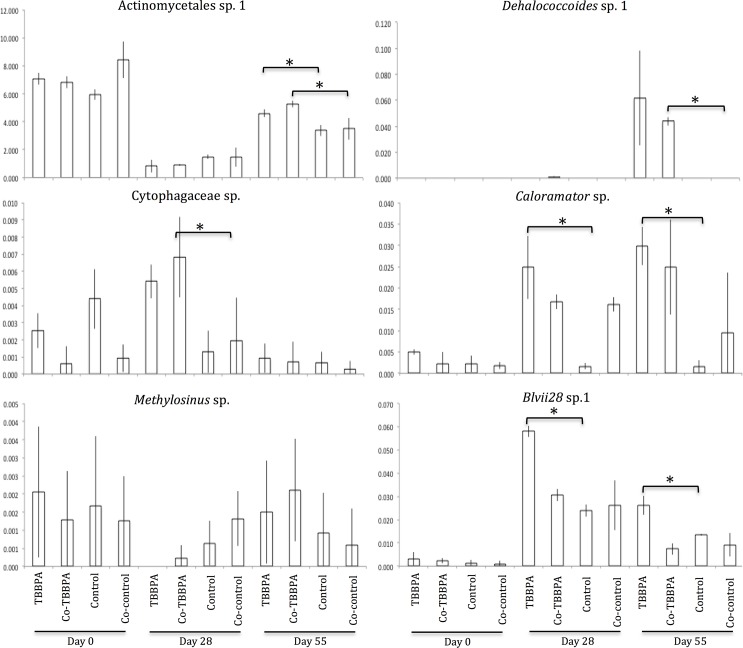
Relative abundance of some of the OTUs having higher abundances in TBBPA-spiked reactors at Day 28 or 55. Error bars represent the standard deviation from the mean. If a bracket and an asterisk are present between two bars, it indicates that the two corresponding samples are significantly different according to a t-test (p ≤0.05) performed for each day separately.

In an attempt to identify the taxa that might be associated with TBBPA degradation, we individually examined each OTU of our dataset and found 58 OTUs that closely affiliated to genera previously described as anaerobic halorespirers[55, 74, 75], or anaerobic degraders of brominated compounds, including TBBPA[39–41, 43, 76]. These taxa included *Pseudomonas*, *Sulfurospirillum*, *Dehalobacter*, *Citrobacter*, *Dehalococcoides*, *Dehalobacterium*, *Acetobacterium*, *Geobacter*, *Anaeromyxobacter*, *Desulfovibrio*, *Comamonas*, *Streptococcus*, *Propionibacterium*, *Methylosinus*, and *Clostridium* species, which all together had a relative abundance of 0.7%. However, not all members of these genera have the capacity to degrade halogenated compounds and not even all strains from the same species have this ability. Therefore, in order to identify taxa possibly metabolizing TBBPA or TBBPA by-products, OTUs that presented a higher relative abundance in TBBPA-spiked reactors at Day 28 and 55 were fetched from our Ion Torrent sequence dataset. Using this approach, 32 OTUs were identified (Table 1), and represented 4.3% of the total microbial community. Among them, 4 were closely related to *Dehalococcoides*, *Desulfovibrio*, *Propionibacterium*, and *Methylosinus* species, while the rest were affiliated to taxa not previously associated with dehalogenating capacity. The relative abundance of a subset of the potential dehalogenating species is presented in Fig 4. Some of these OTUs were found only when anaerobic conditions prevailed in our reactors (i.e., at Day 28 and 55; Fig 4 right panel histograms), while others were detected at Day 0 and Day 28 and/or Day 55 (e.g., Fig 4 left panel histograms), suggesting that both strict and facultative anaerobes may have directly or indirectly been associated with the TBBPA degradation process. This is in line with the fact that both obligate[39] (e.g., *Desulfovibrio* sp.) and facultative[42] (e.g., *Shewanella* sp.) strains have been isolated which have the ability to debrominate organic compounds anaerobically.

**Table 1:** List of the 32 OTUs extracted from our Ion Torrent dataset that were more abundant in TBBPA-spiked than in the control reactors.

Phylum	Class/subdivision	Order	Family	Species/isolate	Characteristics	References
Acidobacteria	Subdivision 4			Ellin6075 (1)	- Isolated from soil	[77]
Actinobacteria	Actinobacteria (1)					
		Actinomycetales (5)				
			Propionibacteriaceae	*Propionibacterium acnes* (1)	- PCE and cDCE degrader	[26, 74]
Bacteroidetes	Bacteroidia	Bacteroidales (3)				
			Rikenellaceae	Blvii28 spp. (2)	- WW sludge lineage. The only isolate is known as anaerobic fermenter of carbohydrates	[78]
	Cytophagia	Cytophagiales	Cytophagaceae (1)			
	Sphingobacteriia	Sphingobacteriales (1)				
Chloroflexi	Dehalococcoidetes	Dehalococcoidales	Dehalococcoidaceae	*Dehalococcoides* sp. (1)	- Degrader of various halogenated, potentially debrominated compounds	[79–81]
Firmicutes	Clostridia	Clostridiales (2)				
			Acidaminobacteraceae	*Acidaminobacter* sp. (1)	- Anaerobic fermenter utilizing amino acids	[82]
			Clostridiaceae	*Caloramator* sp. (2)	- Strictly anaerobic fermenter	[83]
Proteobacteria	α-proteobacteria	Rhizobiales	Hyphomicrobiaceae	*Pedomicrobium* sp. (1)	- Wastewater aerobic oxidizer of Mn and Fe	[84]
			Methylocystaceae	*Methylosinus* sp. (1)	- Methanotrophic, degrading TCE co-metabolically	[74]
	β-proteobacteria	Nitrosomonadales	Nitrosomonadaceae (1)			
	δ-proteobacteria	Desulfobacterales	Desulfobacteraceae	*Desulfonema ishimotonii* (1)	- SRB	[85]
		Desulfovibrionales	Desulfovibrionaceae	*Desulfovibrio* sp. (1)	- SRB capable of degrading TBP	[39]
	γ-proteobacteria	Chromatiales (1)				
		Enterobacteriales	Enterobacteriaceae	*Citrobacter* sp. (1)	- Bacterium with potential for 2,2-DPA dehalogenation	[76]
	Spirochaetes	Spirochaetales	Spirochaetaceae	*Treponema* sp (1)	- H_2_/CO_2_ acetogen	[86]
WWE1	Cloacamonae	Cloacamonales	Cloacamonaceae (1)		- Anaerobic cellulose degraders	[87]
Unknown (2)						

The number of OTUs is indicated between parentheses next to the highest taxonomic level the method used (Uclust; Greengenes database) could allow. Abbreviations: WW = wastewater; PCE = tetrachloroethylene; cDCE = cis-1,2-dichloroethylene; TCE = Trichloroethylene; SRB = Sulfate-reducing bacterium; TBP = 2,4,6-tribromophenol; 2,2-DPA = 2,2-dichloropropionate.

While some taxa within each group had a higher relative abundance on Day 28 compared to Day 55 (e.g., *Cytophagaceae* sp.) suggesting their possible role in the degradation of TBBPA, other taxa were only detected at Day 55 (e.g., *Dehalococcoides* sp., *Actinomycetales* sp. 1, respectively), suggesting that these species might not preferentially degrade TBBPA, but rather may be involved in the degradation of the lower brominated by-products. This result is not completely unexpected as high specificity of debromination capabilities has previously been demonstrated with an isolated *Desulfovibrio* strain, which was able to degrade 2-, 4-, 2,4-, 2,6-, and 2,4,6-bromophenol, but not 3- or 2,3- bromophenol[39]. Within this group of specialists, the dynamic pattern displayed by *Methylosinus* sp. suggests that TBBPA might even be toxic to this taxon. *Methylosinus* sp. is detected at Day 28 only in the control reactors, whereas at Day 55, when TBBPA is completely degraded, *Methylosinus* sp. abundance in the TBBPA-spiked reactors is higher than in the controls. Finally, some taxa had a higher relative abundance only in metabolic reactors (e.g., Blvii28 sp. and *Caloramator* sp.), which might be indicative that the addition of sodium acetate did not create favorable physiological conditions for these taxa.

TBBPA degraders from major bacterial phyla, including Bacteroidetes, Firmicutes, Proteobacteria and Chloroflexi have been isolated. With the exception of 2 OTUs that could not be affiliated to any known phyla, most OTUs identified in the present study belonged to these phyla (Table 1). One of them affiliated to Actinobacteria (‘*Actinomycetales* sp. 1’ in Fig 4), and was one of the overall most abundant OTU in our dataset, with a relative abundance varying from 0.8 to 8.4%. A BLAST search indicates that this OTU closely affiliates with environmental sequences previously detected in sludge during bulking and foaming events such as *Microthrix parvicella*. Actinobacteria species have previously been reported as able to degrade α- and β-Hexachlorocyclohexane[75], suggesting a role of Actinobacteria in TBBPA degradation. *Colaromator* sp. and Blvii28-related OTUs, although never described in the scientific literature as dehalogenating bacteria, also had a significantly higher abundance in metabolic TBBPA-spiked reactors. The role of *Dehalococcoides* sp.-related OTU (Fig 4) in TBBPA degradation is even more likely given that several strains of *Dehalococcoides* have previously been isolated for their ability to degrade a wide range of halogenated compounds[79, 80].

In conclusion, in addition to not altering the overall community composition, as well as the overall performance of the anaerobic reactors (as indicated by the increase of *mcrA* gene copy numbers overtime), TBBPA was efficiently biodegraded. These findings are unexpected considering that some halogenated organic compounds, especially chlorinated and brominated, have been shown to inhibit anaerobic digestion[88]. This study shows that anaerobic sludge microbial communities are resistant and functionally flexible in that the overall community composition was maintained while TBBPA was degraded. TBBPA reductive debromination requires a consortium of species presenting variable degrees of substrate specificity and metabolic preferences. These microorganisms likely interact syntrophically together as well as with non-degrading members of the community, a finding which further explains why co-cultures[43] or enrichment cultures[36, 41] of TBBPA-degrading species degrade TBBPA more efficiently than the few isolated strains obtained to date[18, 40].

## Supporting Information

S1 Fig QIIME commands. (PDF)

S2 Fig OTUs minimum abundance threshold selection. (PDF)

S3 Fig Rarefaction curves. (PDF)

S4 Fig Shannon index comparison between samples groups. (PDF)

S5 Fig Overall taxonomic distribution of Ion Torrent reads. (PDF)

S6 Fig Taxonomic distribution of Ion Torrent reads per sample. (PDF)

S1 Table Ion monitoring details for target compounds and internal standards analyzed by LC-MS/MS. (PDF)

S2 Table Ion Torrent modified 341F and 518R primers. (PDF)

S3 Table Alpha diversity metrics. (PDF)
