# Supplementary material for: Characterization and Adaptation of Anaerobic Sludge Microbial Communities Exposed to Tetrabromobisphenol A
Source: PLoS One. 2016 Jul 27;11(7):e0157622. doi: 10.1371/journal.pone.0157622 (PMC4963083; doi:10.1371/journal.pone.0157622)
Supplement: S1 Fig — (PDF) [file pone.0157622.s001.pdf]

**Figure S1**. QIIME commands used in this study.

- **split_libraries.py -m** *[mapping file name]*.**txt -f** *[raw sequences file name]*.**fasta -q** *[raw quality scores file name]*.**qual -b 18 -z truncate_only -t -M 4 -l 150**

→ Affiliates reads to their respective samples, filters reads based on their mean quality score and length, trims barcodes, adapters, and primers.

- **pick_otus.py -i** *[output file from split_libriries.py]***.fna -s 0.97 -o** *[output directory name]*

→ Clusters reads into operational taxonomic units [OTUs]

- **pick_rep_set.py -i** *[output file from pick_otus.py]***.txt -f** *[output file from spilt_libraries.py]***.fna -o** *[output file name]***.fna**

→ Picks a representative sequence from each OTU

- **assign_taxonomy.py -i** *[output file from pick_rep_set.py]***.fna -m rdp**

→ Affiliates each representative sequence to a taxon

- **make_otu_table.py -i** *[output file from pick_otus.py]***.txt –t** *[output file from assign_taxonomy.py]***.txt -o** *[output otu table name]***.biom**

→ Generates an OTU table

- **filter_otus_from_otu_table.py –i** *[output OTU table from make_otu_table.py]***.biom –o** *[output otu table name]***.biom --min_count_fraction 0.00001**

→ Creates an OTU table in which OTUs with an overall relative abundance below 0.001% are removed

From this OTU table, 4 different workflows were followed:

<u>1- Generates Chao1 individual-based rarefaction curves (Fig S3):</u>

- **multiple_rarefactions.py –i** *[output otu table from filter_otus_from_otu_table]*.**biom –o** *[output directory name]* **-m 1 -x 45000 -s 100 –n 5**

→ Creates rarefied OTU tables

- **alpha_diversity.py –i** *[output directory from multiple_rarefactions.py]* **–o** *[output directory name]* **–m chao1**

→ Calculates the Chao1 index for each rarefied OTU table

- **collate_alpha.py –i** *[output directory from alpha_diversity.py]* **–o** *[output directory name*]

→ Collates all Chao1 indices calculated for each rarefied OTU table

- **make_rarefaction_plots.py –i** *[output directory from collate_alpha.py]* **–o** *[output directory name]* **–m** *[mapping file name]***.txt**

→ Plots the Chao1 individual-based rarefaction curves on a graph

<u>2- Performs a principal coordinates analysis (PCoA) and an analyses of similarity (ANOSIM) on a weigthed UniFrac distance matrix (Fig 2):</u>

- **filter_fasta.py -f** *[output file from pick_rep_set.py]***.fna -o** *[output file name]***.fasta -b** *[output otu*

*table from filter_otus_from_otu_table.py]***.biom**
→ Creates a file containing sequences representative of each OTUs ≥ 0.001% relative abundance; i.e., sequence file identical to the one generated using pick_rep_set.py but without the OTUs having a relative abundance <0.001%

– **align_seqs.py -i** *[output file from filter_fasta.py]***.fasta -t core_set_aligned.fasta -o** *[output directory name]*
→ Aligns representative sequences of each OTU

– **make_phylogeny.py -i** *[output file from align_seqs.py]***.fasta -o** *[output file name]***.tre**
→ Makes a phylogenetic tree with the representative sequences

– **beta_diversity.py -i** *[output otu table from filter_otus_from_otu_table.py]***.biom -m weighted_unifrac -o** *[output directory name]* **-t** *[output phylogenetic tree from make_phylogeny.py]***.tre**
→ Creates a weighted UniFrac distance matrix

– **principal_coordinates.py -i** *[distance matrix generated from beta_diversity.py]***.txt -o** *[output file name]***.txt**
→ Creates a file containing the principal coordinates values to create a PCoA ordination (Fig 2)

– **compare_categories.py --method anosim -i** *[distance matrix generated from beta_diversity.py]***.txt -m** *[mapping file name]***.txt -c** *[factor to test; e.g.,* **Tretament***]* **-o** *[output directory name]*
→ Performs an ANOSIM (analysis of similarity) to statistically test differences between Treatments, Days, or conditions (Fig 2)

<u>3- Calculate alpha-diversity indices and coverage values (Table S2):</u>

– **alpha_diversity.py -i** *[output otu table from filter_otus_from otu_table.py]***.biom -o** *[output file name]***.txt –m chao1,goods_coverage, ,observed_otus,shannon,simpson_e**

<u>4- Compare alpha-diversity metrics using a t-test, and creates boxplots (Fig S4):</u>

– **alpha_diversity.py –i** *[output directory from multiple_rarefaction.py]* **–o** *[output directory name]* **–m Shannon**
→ Calculates the Shannon index for each rarefied otu table

– **collate_alpha.py –i** *[output directory from alpha_diversity.py]* **–o** *[output directory name]*
→ Collates all Shannon indices calculated for each rarefied OTU table

– **compare_alpha_diversity.py -i** *[output file name from collate_alpha.py]***.txt -m** *[mapping file name]***.txt -c** *[name of the group to test]* **-o** *[output directory name]*
→ Performs a t-test between groups and generates boxplots (Fig S4)

<u>5- Creates phylogenetic relative abundance histograms (fig S6):</u>

**summarize_taxa_through_plot.py –i** *[output otu table from filter_otus_from_otu_table.py]***.biom –o** *[output directory name]*
