# Supplementary material for: Characterization and Adaptation of Anaerobic Sludge Microbial Communities Exposed to Tetrabromobisphenol A
Source: PLoS One. 2016 Jul 27;11(7):e0157622. doi: 10.1371/journal.pone.0157622 (PMC4963083; doi:10.1371/journal.pone.0157622)
Supplement: S2 Fig — (PDF) [file pone.0157622.s002.pdf]

Figure S2.

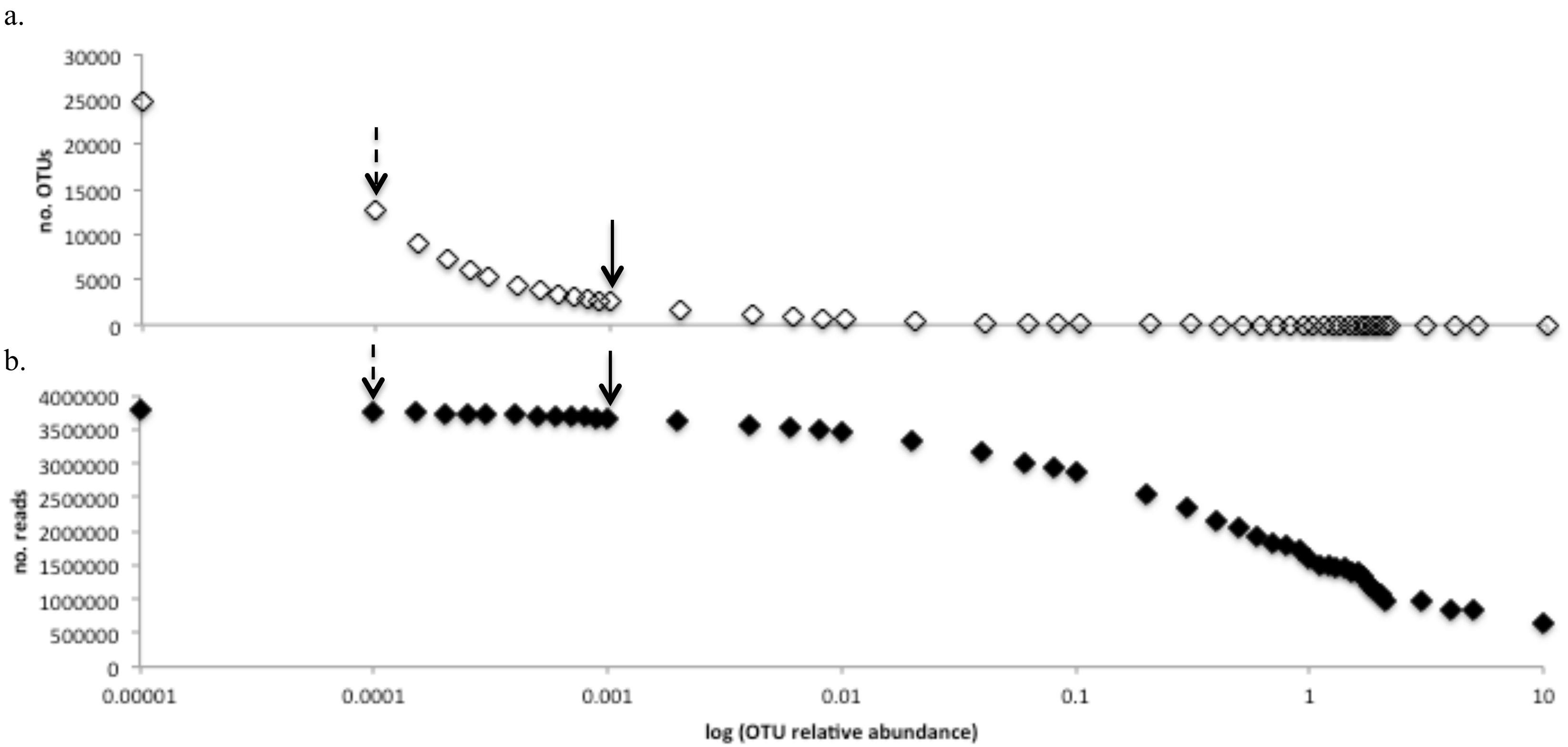

Figure S2. Number of OTUs (a) and reads (b) remaining when OTUs below various relative abundances (x-axis) are removed from the dataset. For example, if 0.0001% is used as a threshold (i.e., all OTUs having a relative abundance below this threshold are removed from the dataset), 3,767,530 reads would remain in the dataset and after clustering these reads at 97% similarity, 12,616 OTUs would be generated (dashed arrows). Using a 0.001% threshold (selected in this study), 3,665,171 reads remained in the dataset, and 2,512 OTUs were generated (plain arrow). The deleted reads were considered to be sequencing errors that would incorrectly inflate the microbial diversity.
