# Supplementary material for: Characterization and Adaptation of Anaerobic Sludge Microbial Communities Exposed to Tetrabromobisphenol A
Source: PLoS One. 2016 Jul 27;11(7):e0157622. doi: 10.1371/journal.pone.0157622 (PMC4963083; doi:10.1371/journal.pone.0157622)
Supplement: S3 Fig — (PDF) [file pone.0157622.s003.pdf]

Figure S3.

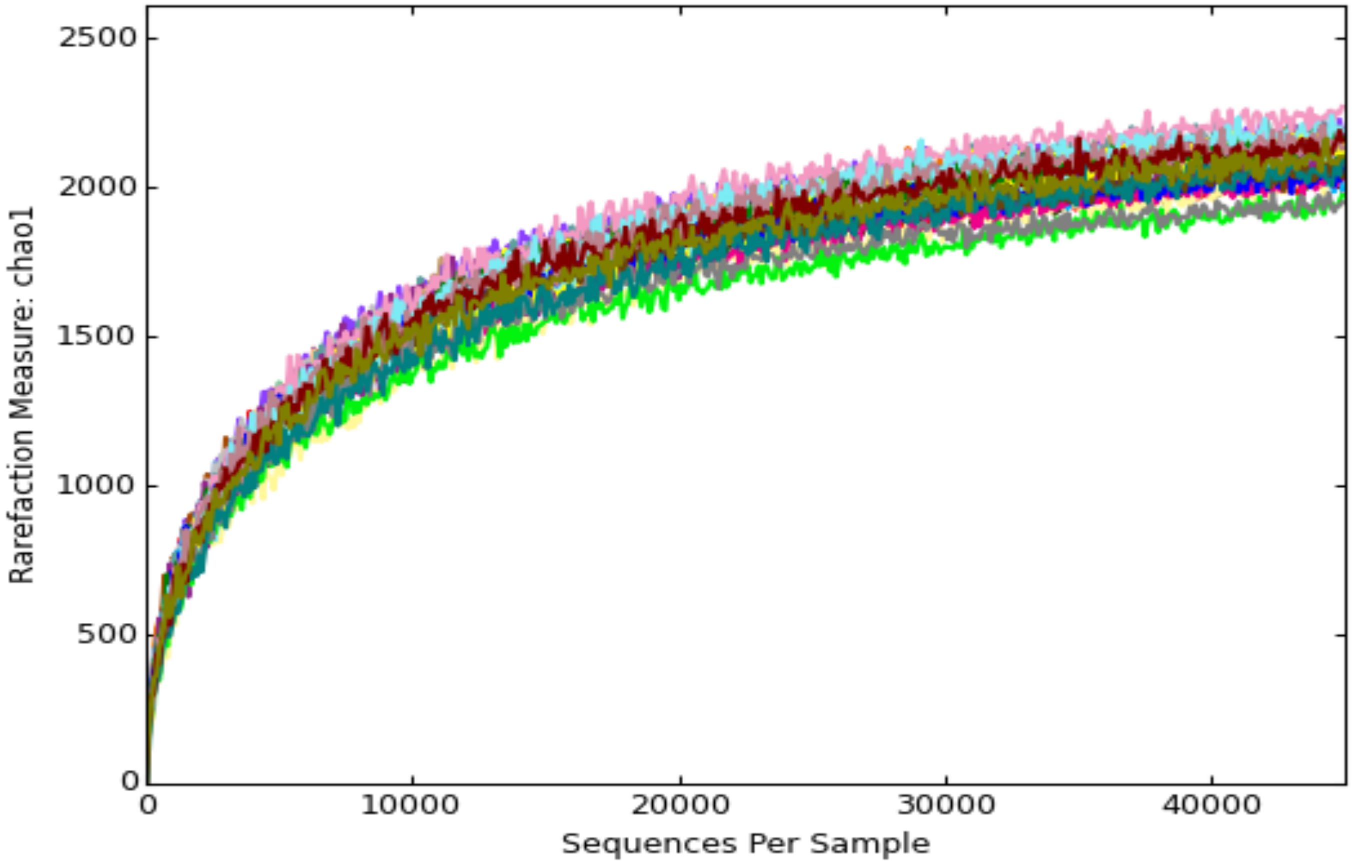

Figure S3. Chao1 rarefaction curves generated for the 36 samples analyzed in this study. The following QIIME scripts were used to generate the graph: **multiple_rarefactions.py –i** [Input otu table name]**.biom –o** [output directory name] **-m 1 -x 45000 -s 100 –n 5; alpha_diversity.py –i** [output directory from previous script] **–o** [output directory name] **–m chao1; collate_alpha.py –i** [output directory from previous script] **–o** [output directory name]**; make_rarefaction_plots.py –i** [output directory from previous script] **–o** [output directory name] **–m** [mapping file name]**.txt**.
