# Supplementary material for: Characterization and Adaptation of Anaerobic Sludge Microbial Communities Exposed to Tetrabromobisphenol A
Source: PLoS One. 2016 Jul 27;11(7):e0157622. doi: 10.1371/journal.pone.0157622 (PMC4963083; doi:10.1371/journal.pone.0157622)
Supplement: S4 Fig — (PDF) [file pone.0157622.s004.pdf]

Figure S4.

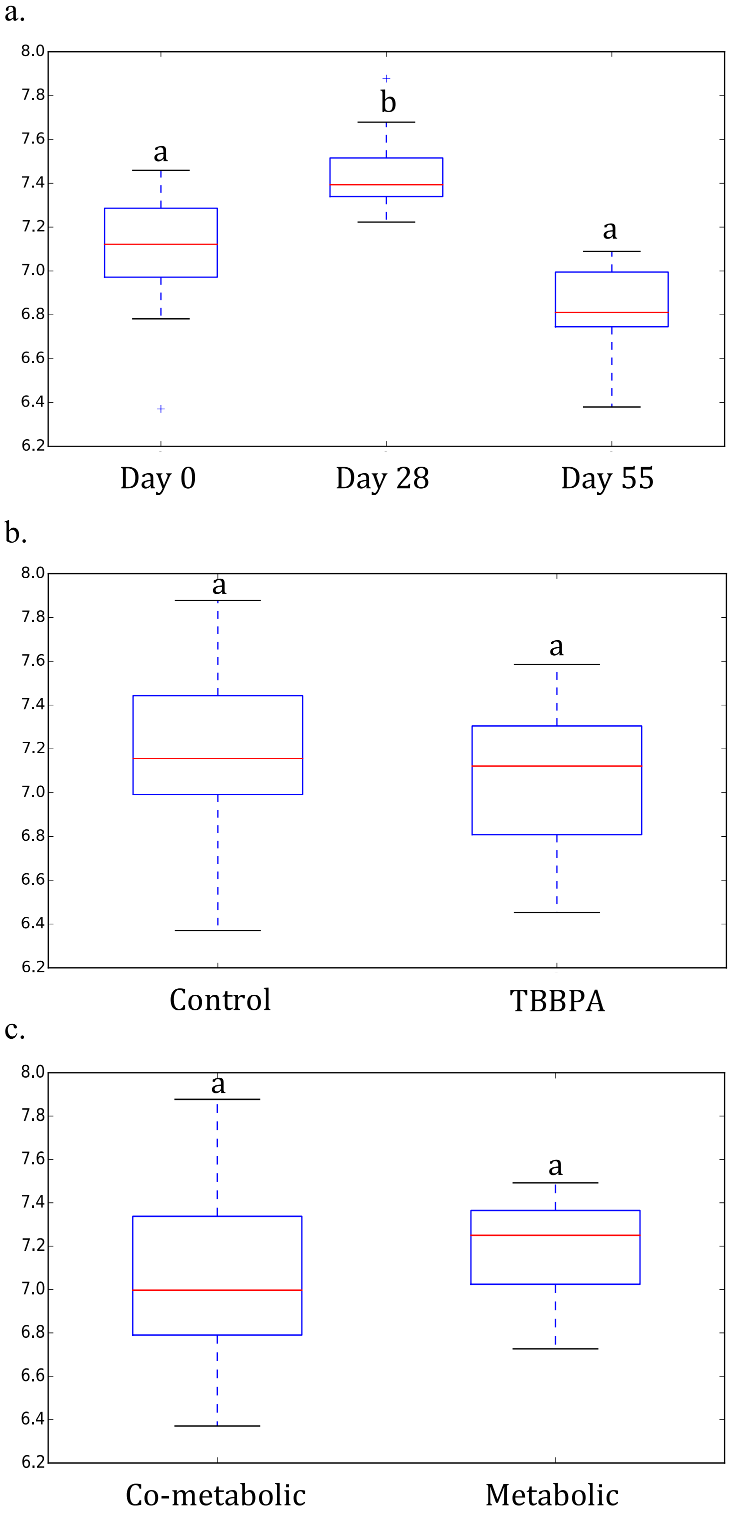

Figure S4. Comparison of the Shannon alpha diversity index between sample groups (a: samples were pooled by day; b: samples were pooled by treatment; c: samples were pooled by metabolism type). The upper and lower boundaries of the box plot illustrate the 25th and 75th percentiles with the median as a solid red line inside the plot. The upper and lower whiskers show the 10th and 90th percentiles with outliers marked in blue outside. Box plots annotated with the same letter are not significantly different (t-test, p threshold=0.05).
