# Supplementary material for: Characterization and Adaptation of Anaerobic Sludge Microbial Communities Exposed to Tetrabromobisphenol A
Source: PLoS One. 2016 Jul 27;11(7):e0157622. doi: 10.1371/journal.pone.0157622 (PMC4963083; doi:10.1371/journal.pone.0157622)
Supplement: S5 Fig — (PDF) [file pone.0157622.s005.pdf]

Figure S5

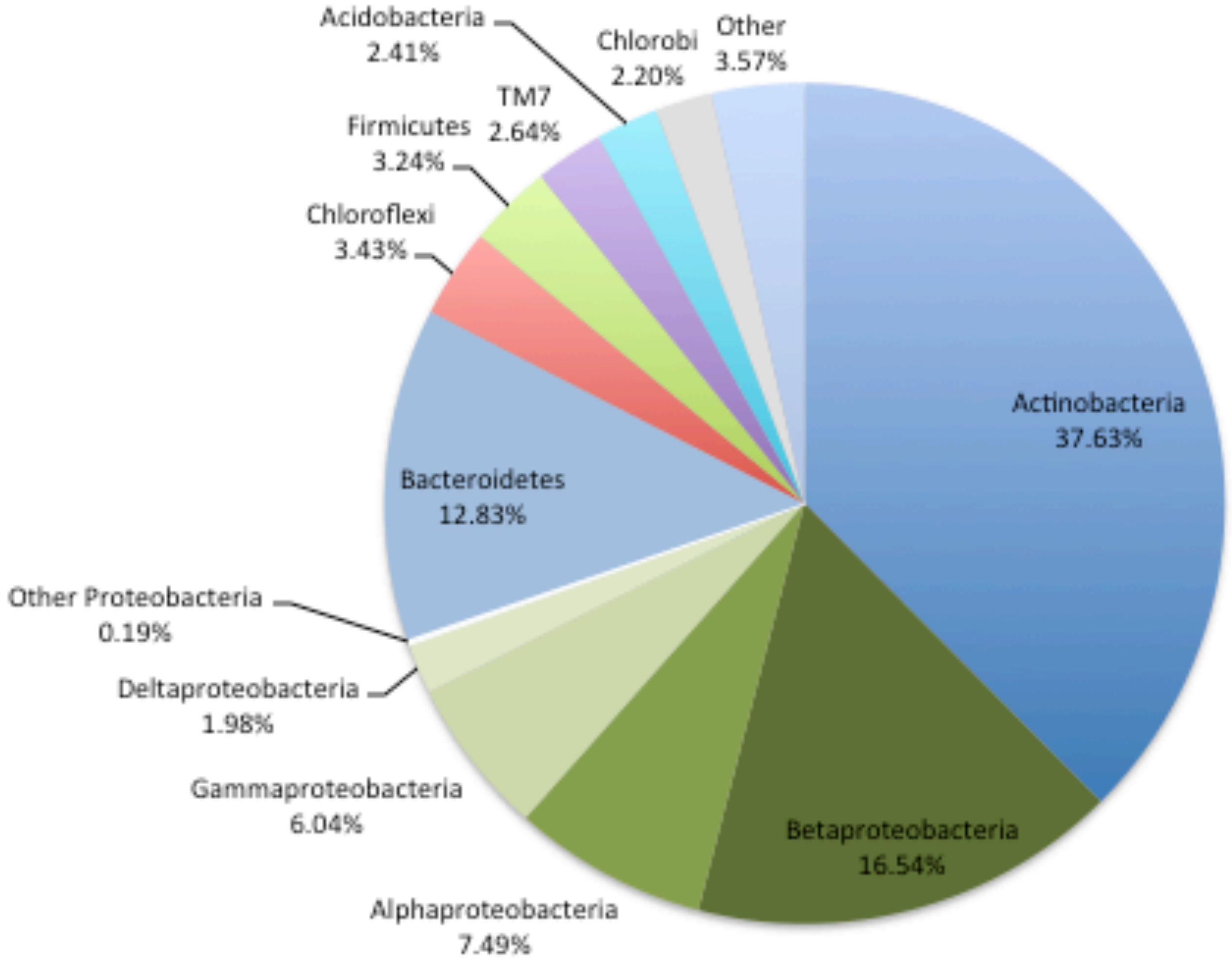

Figure S5. Phylum-level (class-level for the phylum Proteobacteria) distribution and relative abundance of the 16S rDNA V3 region reads obtained in this study.
