# Supplementary material for: Characterization and Adaptation of Anaerobic Sludge Microbial Communities Exposed to Tetrabromobisphenol A
Source: PLoS One. 2016 Jul 27;11(7):e0157622. doi: 10.1371/journal.pone.0157622 (PMC4963083; doi:10.1371/journal.pone.0157622)
Supplement: S6 Fig — (PDF) [file pone.0157622.s006.pdf]

Figure S6.

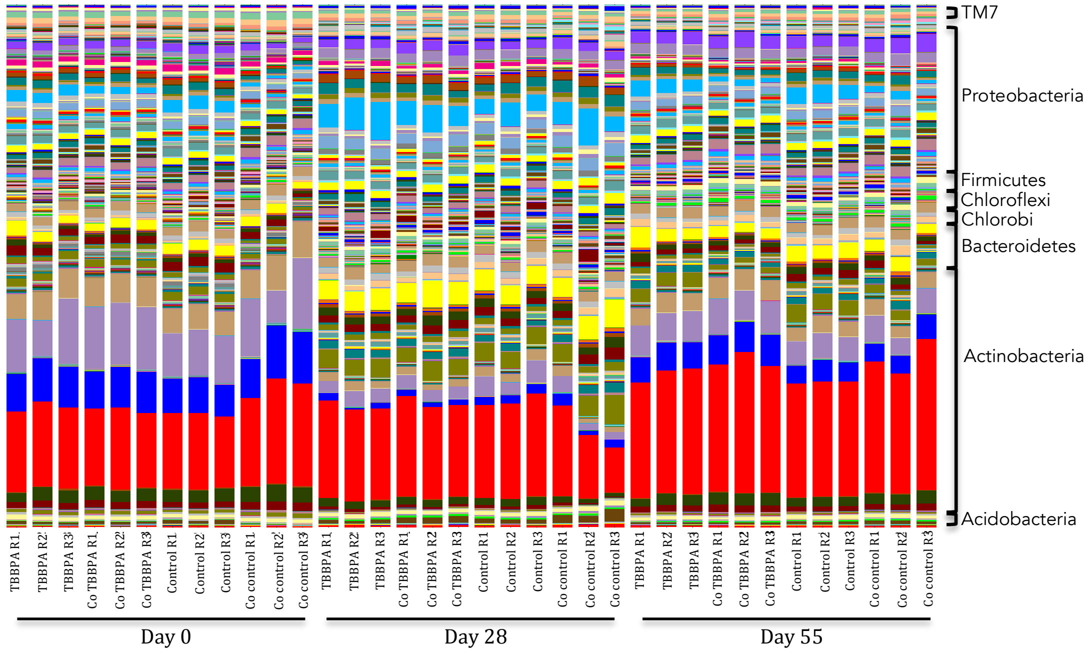

Figure S6. Relative taxonomic distribution of the reads obtained for each sample at the genus level. Each color on the histogram represents a different genus. Only the main phyla are indicated on the right.
