# Supplementary material for: Characterization and Adaptation of Anaerobic Sludge Microbial Communities Exposed to Tetrabromobisphenol A
Source: PLoS One. 2016 Jul 27;11(7):e0157622. doi: 10.1371/journal.pone.0157622 (PMC4963083; doi:10.1371/journal.pone.0157622)
Supplement: S1 Table — (PDF) [file pone.0157622.s007.pdf]

Table S1. Selected ion-monitoring details for target compounds and internal standards analyzed by liquid chromatography-mass spectrometry with negative electrospray ionization.

Compound	Parent Ion (m/z)	Product Ion (m/z)		Collision Energy (V)	S Lens (V)
Target Analytes					
Bisphenol A	227.1	212.1	Q[1]	27	77
	227.1	133.1	C	21	77
Monobromobisphenol A	305.0	79.0	Q	29	107
	305.0	81.0	C	29	107
Dibromobisphenol A	384.9	79.0	Q	41	137
	384.9	81.0	C	41	137
Tribromobisphenol A	462.8	339.9	Q	39	167
	462.8	337.9	C	39	167
Tetrabromobisphenol A	542.5	417.9	Q	40	180
	542.8	419.9	C	40	180
**Primary Internal Standards**					
$^{13}C_{12}$-Bisphenol A	239.2	224.2	Q	21	77
$^{13}C_{12}$-Tetrabromobisphenol A	554.6	428.5	Q	40	180
**Recovery Standards**					
D_8-Bisphenol A	235.0	220.1	Q	21	77
$^{13}C_{12}$-6-hydroxy-2,2,4,4-tetrabromodiphenyl ether	512.7	79.0	Q	18	81

[1] Q: quantifying ion; C: confirming ion.
