# Supplementary material for: Characterization and Adaptation of Anaerobic Sludge Microbial Communities Exposed to Tetrabromobisphenol A
Source: PLoS One. 2016 Jul 27;11(7):e0157622. doi: 10.1371/journal.pone.0157622 (PMC4963083; doi:10.1371/journal.pone.0157622)
Supplement: S2 Table — (PDF) [file pone.0157622.s008.pdf]

Table S2. Ion Torrent modified 241F and 518R primers (5'-3') used in this study to amplify the V3 region of the bacterial 16S rDNA gene. The 36 samples analyzed in this study were processed on two separate Ion Torrent runs. N/A = non applicable.

Primer name	Ion Torrent adapter	Barcode	Spacer	16S RDNA PCR primer
Forward primers				
A341F_BACT_A_21	CCATCTCATCCCTGCGTGTCTCCGACTCAG	GATCTGCGATCC	GT	CCTACGGGAGGCAGCAG
A341F_BACT_A_22	CCATCTCATCCCTGCGTGTCTCCGACTCAG	CAGCTCATCAGC	GT	CCTACGGGAGGCAGCAG
A341F_BACT_A_23	CCATCTCATCCCTGCGTGTCTCCGACTCAG	CAAACAACAGCT	GT	CCTACGGGAGGCAGCAG
A341F_BACT_A_24	CCATCTCATCCCTGCGTGTCTCCGACTCAG	GCAACACCATCC	GT	CCTACGGGAGGCAGCAG
A341F_BACT_A_25	CCATCTCATCCCTGCGTGTCTCCGACTCAG	GCGATATATCGC	GT	CCTACGGGAGGCAGCAG
A341F_BACT_A_26	CCATCTCATCCCTGCGTGTCTCCGACTCAG	CGAGCAATCCTA	GT	CCTACGGGAGGCAGCAG
A341F_BACT_A_27	CCATCTCATCCCTGCGTGTCTCCGACTCAG	AGTCGTGCACAT	GT	CCTACGGGAGGCAGCAG
A341F_BACT_A_28	CCATCTCATCCCTGCGTGTCTCCGACTCAG	GTATCTGCGCGT	GT	CCTACGGGAGGCAGCAG
A341F_BACT_A_29	CCATCTCATCCCTGCGTGTCTCCGACTCAG	CGAGGGAAAGTC	GT	CCTACGGGAGGCAGCAG
A341F_BACT_A_30	CCATCTCATCCCTGCGTGTCTCCGACTCAG	CAAATTCGGGAT	GT	CCTACGGGAGGCAGCAG
A341F_BACT_A_31	CCATCTCATCCCTGCGTGTCTCCGACTCAG	AGATTGACCAAC	GT	CCTACGGGAGGCAGCAG
A341F_BACT_A_32	CCATCTCATCCCTGCGTGTCTCCGACTCAG	AGTTACGAGCTA	GT	CCTACGGGAGGCAGCAG
A341F_BACT_A_33	CCATCTCATCCCTGCGTGTCTCCGACTCAG	GCATATGCACTG	GT	CCTACGGGAGGCAGCAG
A341F_BACT_A_34	CCATCTCATCCCTGCGTGTCTCCGACTCAG	CAACTCCCGTGA	GT	CCTACGGGAGGCAGCAG
A341F_BACT_A_35	CCATCTCATCCCTGCGTGTCTCCGACTCAG	TTGCGTTAGCAG	GT	CCTACGGGAGGCAGCAG
A341F_BACT_A_36	CCATCTCATCCCTGCGTGTCTCCGACTCAG	TACGAGCCCTAA	GT	CCTACGGGAGGCAGCAG
A341F_BACT_A_37	CCATCTCATCCCTGCGTGTCTCCGACTCAG	CACTACGCTAGA	GT	CCTACGGGAGGCAGCAG
A341F_BACT_A_38	CCATCTCATCCCTGCGTGTCTCCGACTCAG	TGCAGTCCTCGA	GT	CCTACGGGAGGCAGCAG
A341F_BACT_A_39	CCATCTCATCCCTGCGTGTCTCCGACTCAG	ACCATAGCTCCG	GT	CCTACGGGAGGCAGCAG
A341F_BACT_A_40	CCATCTCATCCCTGCGTGTCTCCGACTCAG	TCGACATCTCTT	GT	CCTACGGGAGGCAGCAG
A341F_BACT_A_41	CCATCTCATCCCTGCGTGTCTCCGACTCAG	GAACACTTTGGA	GT	CCTACGGGAGGCAGCAG
A341F_BACT_A_42	CCATCTCATCCCTGCGTGTCTCCGACTCAG	GAGCCATCTGTA	GT	CCTACGGGAGGCAGCAG
A341F_BACT_A_43	CCATCTCATCCCTGCGTGTCTCCGACTCAG	TTGGGTACACGT	GT	CCTACGGGAGGCAGCAG
A341F_BACT_A_44	CCATCTCATCCCTGCGTGTCTCCGACTCAG	AAGGCGCTCCTT	GT	CCTACGGGAGGCAGCAG
Reverse primer				
518R_BACT_P1	CCTCTCTATGGGCAGTCGGTGAT	N/A	CC	ATTACCGCGGCTGCTGG
