# Supplementary material for: Characterization and Adaptation of Anaerobic Sludge Microbial Communities Exposed to Tetrabromobisphenol A
Source: PLoS One. 2016 Jul 27;11(7):e0157622. doi: 10.1371/journal.pone.0157622 (PMC4963083; doi:10.1371/journal.pone.0157622)
Supplement: S3 Table — (PDF) [file pone.0157622.s009.pdf]

Table S3. Alpha diversity metrics and coverage values calculated for each sample.

Samples	No. observed OTUs	Estimated OTUs no. (chao1)	Good's coverage	Shannon diversity	Simpson evenness
TBBPA Day 0 R1	1738	2098	99.2%	7.10	0.016
TBBPA Day 0 R2	1884	2162	99.5%	7.32	0.017
TBBPA Day 0 R3	1840	2113	99.4%	6.97	0.013
Co-TBBPA Day 0 R1	1962	2191	99.5%	7.25	0.014
Co-TBBPA Day 0 R2	1715	2098	99.2%	6.99	0.014
Co-TBBPA Day 0 R3	1937	2142	99.6%	7.17	0.014
Control Day 0 R1	1931	2183	99.5%	7.41	0.018
Control Day 0 R2	1860	2126	99.5%	7.29	0.017
Control Day 0 R3	1939	2192	99.6%	7.47	0.018
Co-Control Day 0 R1	1929	2231	99.5%	7.02	0.013
Co-Control Day 0 R2	1755	2004	99.4%	6.79	0.012
Co-Control Day 0 R3	1746	2077	99.4%	6.39	0.010
TBBPA Day 28 R1	2008	2254	99.6%	7.38	0.014
TBBPA Day 28 R2	1945	2268	99.4%	7.38	0.015
TBBPA Day 28 R3	1952	2238	99.5%	7.30	0.014
Co-TBBPA Day 28 R1	2117	2329	99.7%	7.44	0.012
Co-TBBPA Day 28 R2	2177	2382	99.8%	7.29	0.013
Co-TBBPA Day 28 R3	2182	2377	99.7%	7.61	0.014
Control Day 28 R1	2102	2321	99.7%	7.49	0.014
Control Day 28 R2	2210	2377	99.8%	7.52	0.013
Control Day 28 R3	2239	2402	99.8%	7.26	0.011
Co-Control Day 28 R1	2140	2354	99.7%	7.38	0.014
Co-Control Day 28 R2	2051	2333	99.6%	7.70	0.022
Co-Control Day 28 R3	1719	2037	99.1%	7.88	0.039
TBBPA Day 55 R1	1953	2311	99.5%	6.82	0.010
TBBPA Day 55 R2	2105	2390	99.7%	6.79	0.009
TBBPA Day 55 R3	2172	2350	99.8%	6.76	0.008
Co-TBBPA Day 55 R1	2056	2314	99.6%	6.83	0.008
Co-TBBPA Day 55 R2	2104	2347	99.7%	6.48	0.007
Co-TBBPA Day 55 R3	2186	2385	99.8%	6.78	0.008
Control Day 55 R1	2227	2423	99.8%	7.06	0.009
Control Day 55 R2	2318	2461	99.8%	7.06	0.009
Control Day 55 R3	2236	2393	99.8%	7.11	0.009
Co-Control Day 55 R1	2176	2367	99.7%	6.89	0.008
Co-Control Day 55 R2	2076	2358	99.7%	7.01	0.009
Co-Control Day 55 R3	1630	2080	99.1%	6.39	0.008
